# Exploring cooperation among social pathogens: a computational perspective

**DOI:** 10.1093/femsre/fuag007

**Published:** 2026-02-24

**Authors:** Andrea S Ramirez-Mata, Cameron Browne, Ryan S Doster, Marco Salemi, Brittany Rife Magalis

**Affiliations:** Emerging Pathogens Institute, University of Florida, Gainesville, FL, 32610, United States; Department of Pathology, Immunology and Laboratory Medicine, University of Florida, Gainesville, FL, 32610, United States; Department of Mathematics, University of Louisiana Lafayette, Lafayette, LA, 70504, United States; Department of Medicine, Division of Infectious Diseases, University of Louisville School of Medicine, Louisville, KY, 40202, United States; Department of Microbiology and Immunology, University of Louisville School of Medicine, Louisville, KY, 40202, United States; Center for Predictive Medicine, University of Louisville School of Medicine, Louisville, KY, 40202, United States; Emerging Pathogens Institute, University of Florida, Gainesville, FL, 32610, United States; Department of Pathology, Immunology and Laboratory Medicine, University of Florida, Gainesville, FL, 32610, United States; Center for Predictive Medicine, University of Louisville School of Medicine, Louisville, KY, 40202, United States; Department of Biochemistry and Molecular Genetics, University of Louisville School of Medicine, Louisville, KY, 40202, United States

**Keywords:** pathogen, social, cooperation, co-evolution, genomics, phylogeny

## Abstract

Once centered on animal social behavior, investigations into cooperation have expanded across the tree of life to include micro-organisms such as bacteria and viruses. Cooperative interactions are now understood to drive evolutionary dynamics within and between numerous microbial species and communities, including pathogen adaptation to and persistence in new hosts and environments. Identification and characterization of the underlying mechanisms of cooperation offer innovative opportunities for therapeutic interventions targeting difficult-to-treat pathogens through disruption of interactive networks. The current gold standards for evaluating micro-organismal cooperation often rely on assessing coordinated changes of phenotypic traits and the genetic and environmental factors that can affect them. Among these approaches, *in vitro* methods are labor-intensive, time-consuming, and often fail to replicate the natural microenvironment. Computational methods applied *in vivo* offer scalability and applicability but often require prior knowledge of metabolic pathways, restricting their use to bacterial systems. In contrast, sequence- and phylogeny-based frameworks can extend to viral datasets, though are typically con- strained by smaller sample sizes and incomplete annotations. Herein we focus on existing computational approaches used in identifying and/or characterizing cooperation and detail their advantages and limitations in shaping our understanding of cooperative pathogens.

## Teaser

Computational approaches are increasingly used to study microbial and viral cooperation, offering scalable alternatives to traditional methods, though current tools remain limited in scope, especially for viruses and complex *in vivo* systems.

## Introduction

Social behavior is broadly defined as the interactions that occur among individuals (Dukas and Bailey 2024). Those interactions that result in reciprocal benefits can be considered cooperative and play an essential role in the survival and evolution of numerous species (Dale et al. 2020, Preussger et al. 2020). Here, we use the term cooperation to refer to behaviors that enhance the fitness of the individuals (or populations) involved and that evolve due to the advantages they confer (West et al. 2007). By contrast, behaviors that incur a net fitness cost to the actor while benefiting others, are considered altruistic (Sachs et al. 2004). While social behaviors may be influenced by more antagonistic interactions, or competition, (Madgwick et al. 2018), these interactions have been discussed at length elsewhere (see Baishya and Wakeman 2019, Goncalves et al. 2020, Leeks et al. 2023), whereas cooperation remains relatively underexplored in the context of micro-organisms (Sachs and Hollowell 2012, Sanchez and Gore 2013, Wu et al. 2023).

Within the animal kingdom, a vast variability exists in terms of degree and type of cooperative behavior (Messias et al. 2016). Even within a single-species population, this behavior can be complex, involving multiple moving parts, and dynamic in response to environmental shifts (Komdeur 1992, Field et al. 2006). The scope of complexity widens when one considers the cost often ascribed to an individual in cooperative behavior, which seemingly contradicts the fundamental tenets of natural selection (Sachs et al. 2004, Harcombe 2010). After all, why would an organism engage in the benefit of others when it can exploit available resources to the full extent without reciprocating? Despite this apparent contradiction, cooperation is widespread across diverse biological systems, extending beyond the animal kingdom, and can occur both within populations and between genetically distinct populations or species. Bacteria and fungi are among the more popular of the micro-organisms on the list of cooperative biosystems under investigation (Goncalves et al. 2020, Sadiq et al. 2021, Lee et al. 2022, Zhao et al. 2022), though amoeba (Medina et al. 2024) and viruses are gaining traction (Van den Bergh et al. 2018, Phipps et al. 2020). To cope with rapidly changing environments and host defenses, these microbes may rely on an array of resources and mechanisms. The sheer diversity and complexity of currently characterized cooperative mechanisms underscores the need to develop tools equipped to identify novel interactions that promote survival as they emerge. Driven by this need, we examine the methodological approaches used to study microbial cooperation while providing a broader perspective on the phenomenon itself.

In this review, we will highlight recent discoveries of cooperative behaviors in both pathogenic and non-pathogenic micro-organisms, emphasizing their implications for microbial ecology and evolution and development of related tools. For example, one important area of cooperative investigation is the modern biomedical and agricultural applications of engineered microbes (e.g. bioremediation). Deployment of genetically modified organisms (GMOs) in complex open environments can result in ecological interactions that can have unexpected consequences for containment and function (Pantoja Angles et al. 2022), which is why their deployment is subject to mandated biological containment measures. Although effective in controlled laboratory settings, these safeguards can lose efficacy in complex environments due to fluctuating conditions, microbial diversity, mutation, or unanticipated genetic interactions (Kim and Lee 2020, Arnolds et al. 2021, Zhu et al. 2023, Varma et al. 2025). As GMOs are used more often in open environments, it is becoming increasingly important to understand how they interact with the native microbial communities around them (Arnolds et al. 2021). Understanding cooperative mechanisms is also particularly relevant for pathogens, where microbial survival directly impacts host health, be it humans or the plants and animals on which we rely. In order to identify the emergence of cooperativity and evaluate its impacts on human health, it is necessary to unravel the underlying factors and mechanisms in a timely, biologically relevant, and cost-effective manner.

Laboratory methods currently employed to investigate microbial cooperation, such as *in vitro* physiological and biochemical assays, are laborious and time-consuming [for a more in-depth discussion, see (Rajapaksha et al. 2019, Franco-Duarte et al. 2019)]. Additionally, they may not accurately mimic the natural social dynamics (Giraffa 2004), such as interactions with more complex multicellular organisms (Franzosa et al. 2015, Gamalero et al. 2022) or even other species that may not be cultivable in the lab. The limited ability to cultivate reliant species introduces a bias if the difficulties associated with culturing are driven by mutually beneficial interactions essential for the growth of the micro-organism under investigation (Foster and Bell 2012). Without specialized tools for evaluating specific interactions in changing microbial communities *in vivo*, the larger impact of social dynamics within and among microbial communities will remain speculative. There exist approaches aimed at evaluating cooperative behavior that can be applied to sampled microbes from their original environment; however, limitations described in this review, which include requirement of extensive prior knowledge, incompatibility with viruses, and applications to large datasets, posit a need to develop new techniques. New avenues for the detection, investigation, and comprehension of the genetic foundations of cooperation have been emerging in the fields of molecular genetics, comparative genomics, and evolutionary theory that we propose are highly applicable in this arena (Wade 2007). Some of these strategies are not entirely novel, as they have been employed in other domains of life, owing to the existence of cooperation at virtually every biological level. We end this review by addressing how both new and existing approaches address current research gaps and may even be combined to identify novel cooperative mechanisms and test hypotheses of microbial community participation in biofilm formation, immune evasion, and within-host evolution.

## Natural selection and ecological interactions: essentials for adaptive evolution

Engaging in cooperation, even at the cost of self-preservation (often described colloquially as altruistic behavior), is an emerging property that may actually be best explained in light of selection. In other words, cooperation is likely to emerge when rewards for an individual bearing a cooperative gene surpass the benefits of acting alone (Fletcher and Doebeli 2008). For example, the behavior may confer an indirect selective advantage to a seemingly selfless individual by facilitating the transmission of its genetic material carried by its relatives (Hamilton 1964). This type of family-focused cooperation, termed kin selection, has been primarily associated with animal species—e.g. bee colonies (Hall and Goodisman 2012, Naeger et al. 2013)—but can also be found among micro-organisms (Smith et al. 2016, Brückner et al. 2020, Simonet and McNally 2021, Belcher et al. 2022). While kin selection is relatively common in nature, there are also studies exposing the establishment and support of cooperation among less closely related individuals (Clutton-Brock 2009, Riehl 2013). In this context, cooperative behavior(s) may arise for the purpose of obtaining mutual gains, sharing of common interests, or retribution against cheating behavior (Buckling et al. 2007), ultimately increasing population survival rates (Cremer et al. 2019) in the face of shared selection pressures. Together, these principles establish that cooperative behavior can be favored by selection and stabilized through ecological and evolutionary mechanisms that leave measurable signatures at the genetic and population levels, signatures that form the basis for many of the computational approaches reviewed below.

Adaptation of a species in the face of selective challenges often depends on: first, the type of imposing selection pressure within the environment (Vinton et al. 2022) and second, the intricate factors involved in the adaptive response (Lafuente and Beldade 2019). Selection pressures come in a variety of flavors, emerging in the form of changes in both the biotic and abiotic environment (Donihue and Lambert 2014). Some of these changes include forced migration and colonization of a new habitat (Olson-Manning et al. 2012), the emergence of new host immune defenses (Nijmeijer and Geijtenbeek 2019), and natural and synthetic antimicrobial agents (Hansen et al. 2011, King et al. 2015). At its core, natural selection operates on the premise of fitness, which quantitatively describes an organism’s reproductive competence and its potential to transmit its genetic material (Lambert and Kussell 2015). Maximizing fitness entails evaluating the benefits of current reproduction in relation to the potential drawbacks on future reproductive success (Linden and Møller 1989). While individuals seek to maximize their individual fitness, even if such optimization imposes costs to other individuals or their group (West et al. 2015), the survival of the population can become a decisive factor in determining survival of the individual. Understanding how the precise balance between individual and population fitness is achieved in the face of environmental changes, often at a high cost to certain individuals (classic evolutionary theory) (Li et al. 2022), has been a challenge in evolutionary biology and extends into microbial ecology and evolution.

It is widely acknowledged that micro-organisms such as bacteria are able to readily adapt to environmental changes through regulation of their metabolic activities, and production of secondary metabolites, but also formation of biofilms (Baishya and Wakeman 2019). Bacteria as part of biofilms are able to engage in communication with each other through the utilization of chemical signaling molecules, and the information provided by these signals plays a vital role in coordinating function and behavior as a group in response to environmental changes (Rana et al. 2021). This cell-to-cell communication, termed quorum sensing (QS), is a regulatory mechanism used to modulate a vast array of genes. QS is used, for example, by the pathogenic bacteria *Salmonella enterica* to regulate motility and to form a protective biofilm in the presence of harsh environments, which can ultimately lead to clinical problems such as bacteraemia, gastroenteritis, and enteric fever (Rana et al. 2021, Dawan et al. 2022). For example, bile salts encountered by *S. enterica* during its transition from the intestine to the gallbladder are a natural bactericidal agent that induces oxidative stress (Walawalkar et al. 2016). In response, *S. enterica* serovar Typhi is capable of up-regulating superoxide dismutase (SOD) and catalase through a QS system activated by autoinducer-2 (AI-2), generating a more beneficial, antioxidant environment. Processes that are coordinated through QS, such as the antioxidant response, secretion of virulence factors, and even bioluminescence, have proven inefficient when executed by an individual bacterial cell compared to when carried out in the context of a bacterial community (Fuqua et al. 2001, Montgomery et al. 2013, Papenfort and Bassler 2016). This inefficiency is partly because it is simply too costly for a single cell to express all of the genes involved in these and other processes required for survival. Instead, the numerous gene products regulated by QS can be communally shared among members of the population, representing public goods (West et al. 2006). This cooperative strategy can also come at a cost to the actor, however, as some group members may contribute more than others, while other members might try to benefit without producing or expending any resources (Preussger et al. 2020).

The bacteria *P. aeruginosa* can similarly form biofilms that confer protection from oxidative stress, phagocytosis (Moradali et al. 2017), and the host immune response (Ciofu and Tolker-Nielsen 2019). A widespread bacterium found in environmental and mammalian hosts (Crone et al. 2019), it causes infections of the blood, urinary tract, and skin (Wood et al. 2023), and is a major contributor to acute and chronic respiratory disease in cystic fibrosis (CF) and advanced COPD patients (Crone et al. 2019, Morin et al. 2021). Samples of this bacterial species isolated from clinical and environmental settings have been described to be particularly susceptible to cheaters (Asfahl and Schuster 2018, Chen et al. 2019, Smith et al. 2019). Cheating in this and other microbial populations often arises through selfish mutations that confer a growth advantage, such as enhanced uptake of peptides, but can ultimately lead to population collapse and even death—a phenomenon also known as “tragedy of the commons” (Lynn and De Leenheer 2019). However, in this specific case, the cheating *P. aeruginosa* population can be regulated by policing mechanisms, such as the secretion of cyanide (pleiotropically linked to protease production) by the more resistant wild-type (WT) cells, directed by cell-cell signaling systems (West et al. 2006). When opportunities for individuals to engage in cheating behavior can be diminished or even eliminated, the reproductive success of individuals improves by increasing the productivity of the group. Neighbors can thus unite through a common interest of repressing the cheaters via differing mechanisms (punishment, sanctions, policing), selecting for the act of cooperation itself (West et al. 2006).

In viruses, cheaters can emerge in the form of defective interfering genomes or particles (DIPs). DIPs can arise spontaneously during infection through mutations that delete genes encoding replication enzymes, capsid proteins, or host-modulating factors. These deletions render DIPs unable to replicate efficiently on their own, yet they exploit co-infecting WT viruses to boost their own replication, often reducing the fitness of the latter (Rezelj et al. 2018, Leeks et al. 2021). This evolutionary logic has now been leveraged experimentally: a naturally arising human immunodeficiency virus (HIV) deletion variant was engineered to enhance its conditional transmission, creating what the authors call a “therapeutic interfering particle” that replicates only in the presence of the WT virus. By competing for viral proteins and packaging machinery, this engineered cheater spreads alongside the WT virus while suppressing its replication. A single administration of this engineered cheater was reported to persistently suppress viral replication, reduce disease severity, and extended survival in non-human primates without evidence of viral escape (Pitchai et al. 2024).

## Mutations can alter the steady state of a population

Mutations can strongly influence population steady-state dynamics. As illustrated by the *P. aeruginosa* example discussed above, selfish mutations in cheater lineages can enhance peptide uptake and confer a short-term growth advantage, potentially collapsing the population if left unchecked (West et al. 2006, Lynn and De Leenheer 2019). Interestingly, in the case of *P. aeruginosa*, populations often reach an equilibrium wherein cheaters are able to coexist with their WT cooperative counterparts (Asfahl et al. 2015, Smith et al. 2019). The dynamic equilibrium between cheater and cooperator phenotypes, maintained by such policing mechanisms, prevents collapse and ensures population stability.

**Figure 1:**
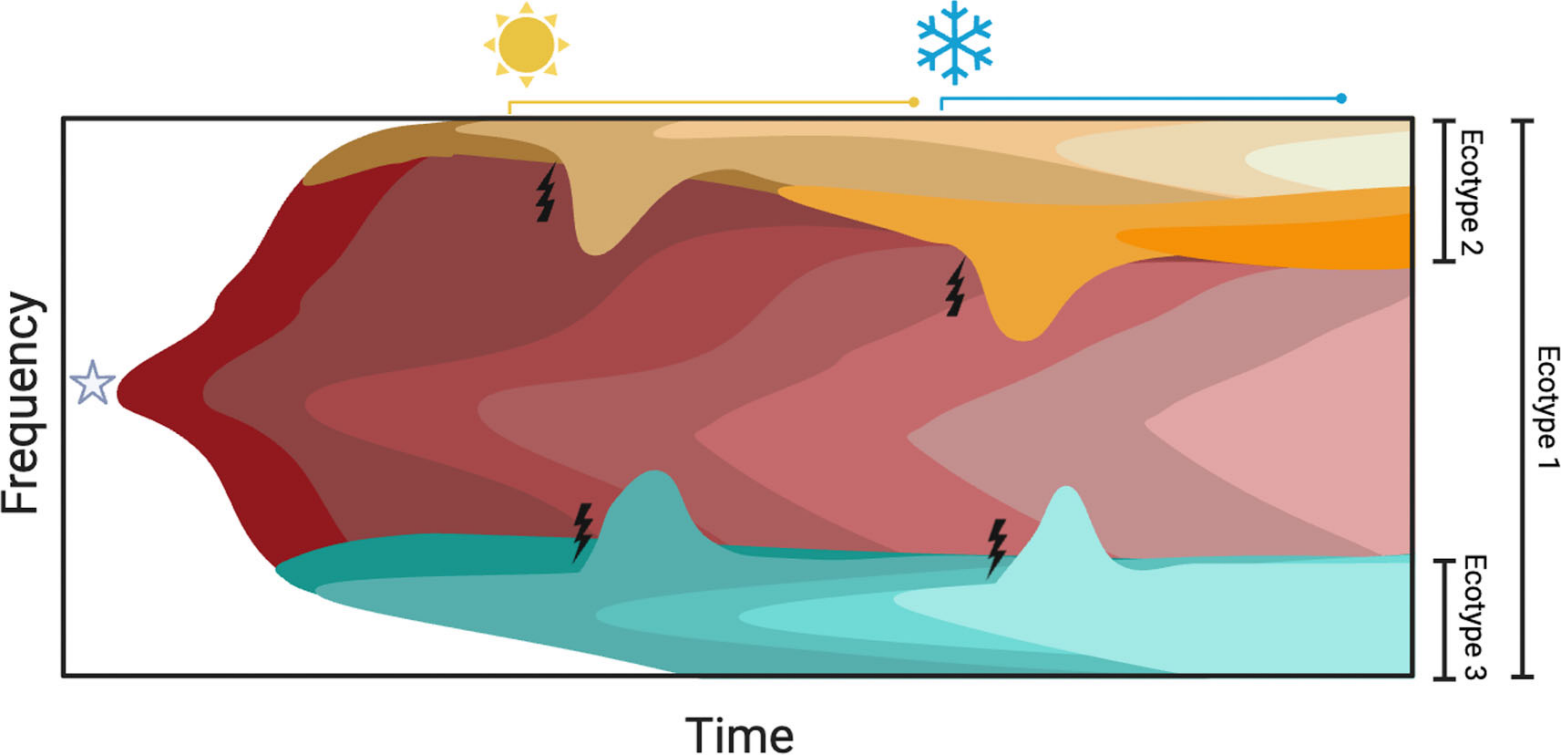
Maintenance of a cooperative biosystem in response to environment-mediated emergence of cheaters through compensatory mutations. This figure highlights how cooperative lineages can persist, despite the emergence of cheaters, through compensatory mutations. Frequencies (*y*-axis) and mutational dynamics of each haplotype within the population over time (*x*-axis). Each haplotype is represented by a different primary color. The gray star indicates the introduction of the representative micro-organism into the current environment. The sun and snowflake on top denote seasonal changes in temperature as changing selective pressures, though can represent any significant change in the environment. Mutations impacting haplotype frequencies are represented by lightning bolts.

Because cheaters often exhibit both a differential phenotype and genotype from cooperative organisms within a population, they can be identified by monitoring their relative frequency within the population (Allen et al. 2016). Their contribution to the population can be characterized by detecting the mutation(s) (Chen et al. 2019) associated with the observed frequency variation and the compensatory mutation(s) required to once again stabilize the population (Robitaille et al. 2020, Traverse et al. 2012) (Fig. 1). These mutations can then be used to trace environmental changes (e.g. seasonal changes in weather, Fig. 1) that may have triggered their emergence and disruptions in cooperative behavior. As a case study, Traverse et al. (Traverse et al. 2012) cultured over one thousand generations of *Burkholderia cenocepacia*, generated by daily adherence and dispersal from a plastic bead, to uncover the evolutionary influences and mutations that allow this bacteria to adapt and diversify as a biofilm associated with cystic fibrosis. They observed, through mutational patterns, a consistent tendency of biofilm generalists to become specialists, with distinct functionalities. For more examples of this behavior, see (Xu et al. 2022, Meijenfeldt et al. 2023). Those mutational patterns showcased the existence of multiple adaptive alleles concentrated at a handful of genetic loci (Traverse et al. 2012). Intriguingly, mutations that disrupted the equilibrium among these specialist subpopulations were observed, followed by compensatory mutations acting to recover this equilibrated state. This finding proposed a sophisticated and previously overlooked interconnectedness of the otherwise unrelated set of genes involved (Kessler and Kim 2022). Additionally, metagenomic data collected from five biofilm lineages that evolved independently unveiled a surprising degree of mutational convergence. Similar mutations were even detected in *Pseudomonas* isolates from cystic fibrosis lung samples, illustrating shared evolutionary pathways among chronic bacterial infections of the lungs (Traverse et al. 2012).

Mutations can also create a favorable steady state. One example is influenza virus, for which mutations in one protein can transform a relatively homogeneous population into that of a coexistence of multiple viral genotypes at stable frequencies over time. The influenza virus genome encodes for two surface proteins referred to as hemaglutinin (HA) and neuraminidase (NA), which function in cell entry and exit, respectively (Du et al. 2019). In clinical H3N2 samples passaged in cell culture, WT aspartic acid (D) at amino acid position 151 in NA has been observed to mutate to glycine (G). The reverse was also observed, wherein D151 emerges from a pure population of G151. Over time, these mutations lead to a mixed population which can stably maintain both variants at steady frequencies (Xue et al. 2016), resembling the *Burkholderia* example described above. The functional consequences of this mutational behavior are discussed in more depth in the section Co-variation and co-evolution as indicators of social behavior.

The interplay between genotypic adaptations and population dynamics, as observed in H3N2 and *Pseudomonas*, highlights the intricate balance required to sustain cooperative behavior. These findings emphasize that population stability is not only influenced by mutations within individual organisms but also by their ecological contexts (i.e. cell culture or host), and even by the spatial structure in which these populations exist. These dynamics are also important examples of how mutations can produce measurable population signatures relevant to inferring cooperative behavior.

## Population structure enhances cooperativity

Bacterial biofilms often exhibit spatial heterogeneity, which can facilitate resource allocation and communication (i.e. QS) between neighboring cells (Platt 2023). In other words, public goods generated by an individual primarily favor the immediate surrounding individuals (Pande et al. 2015). Hence, individuals do not engage in interactions with every other individual but are limited to their neighbors, reflective of an underlying network of connections (Liu et al. 2016). The organization of these networks emerges through evolutionary processes, wherein the interplay of environmental factors, physical constraints, and the population dynamics collectively contribute (Traverse et al. 2012, Van den Bergh et al. 2018). In fact, single-species bacterial biofilms often do not start out as populations, but arise as a result of proliferation of a single bacterium (Melaugh et al. 2016). In multi-species biofilms, instead, different bacterial species join forces, often due to metabolic interdependence or environmental conditions that favor communal living (Pande et al. 2015, Liu et al. 2016). Prime examples of multi-species biofilms include dental plaques, which can lead to cavities and gum infections, potentially resulting in tooth decay and tooth loss (Ray 2022). Species like *Actinomyces naeslundii* and *Streptococcus gordonii* have limited ability to form biofilms independently in saliva, but they are able to reciprocally benefit from metabolic byproducts when co-cultured (Liu et al. 2016).

Beyond surface-associated biofilms, micro-organisms can also organize into spatially structured communities in fluid environments, creating biofilm-like microhabitats that shape the efficacy of pollutant breakdown in bioremediation. During an oil spill, for example, oil droplets form that act as sites for microbial colonization (Quigg et al. 2016). Chemical dispersants, applied as remediation, break the oil into even smaller droplets that can be more readily colonized by these micro-organisms. Doyle et al. (2018) conducted controlled experiments to examine how the chemical dispersant Corexit®affects the colonized microbial community and, ultimately, oil dispersion. The group observed that microbial diversity was significantly reduced among the chemically dispersed oil (as compared with oil-only droplets), dominated by bacteria specialized in breaking down simpler hydrocarbons. Importantly, the dispersed oil harboring this low-diversity community appeared to slow the normal progression of oil breakdown and favored the downward transport of oil, rather than its complete removal at the surface, referred to as marine oil “snow.” This snow-like transport is the result of oil droplet aggregation, which is due in large part to the microbial secretion of exopolymeric substances (EPS) (Doyle et al. 2018). In other words, chemical dispersants can negatively impact the fate of oil transport and persistence following a spill through disruption of microbial diversity and increased microbial cooperativity (Quigg et al. 2016). This finding alone underscores the need for bioremediation strategies that explicitly account for microbial cooperation, as cooperative mechanisms can strongly shape oil degradation trajectories and downstream environmental outcomes.

Another example of manipulation of cooperative interactions through spatial structure is horizontal gene transfer (HGT), the transfer of genetic material between micro-organisms. The likelihood of HGT increases with increased spatial proximity between donor and recipient (Emamalipour et al. 2020). Since the earliest stages of life’s evolution, the availability and acquisition of diverse DNA molecules within cells has profoundly shaped every organism (Rodríguez-Beltrán et al. 2021). Gene transfer, integral to this early microbial evolution, remains particularly prominent in bacteria. Genes obtained via HGT can increase fitness and allow bacteria to occupy ecological niches unattainable through mutation alone (Lee et al. 2022). HGT plays a critical role, for example, in spreading antibiotic resistance, virulence factors, and traits that shape human infections (Zhou et al. 2021). Bacteria can acquire antibiotic resistance via HGT specifically with the acquisition of mobile elements such as plasmids, which are self-replicating DNA elements that co-exist with chromosomes and have been key in facilitating prokaryotic evolution (Rodríguez-Beltrán et al. 2021). For example, in *Escherichia coli*, pCT plasmids carrying the penicillin- and third-generation cephalosporin-resistance gene blaCTX-M-14 impose minimal fitness costs, allowing plasmid-bearing cells to coexist with non-carriers at low but stable frequencies. However, the fitness advantage of carrying the plasmid is higher when plasmid-bearing bacteria are rare Dimitriu et al. 2019, emphasizing the importance of recognizing minor subpopulations in terms of functional contribution to the overall structured population Dimitriu et al. 2019, Amanatidou et al. 2019.

Similar to biofilms, viral populations can exhibit tremendous heterogeneity, forming what is often referred to as a quasispecies (Domingo et al. 2012). Virus populations that qualify as a quasispecies can exhibit natural self-organization that may enable evolution into more complex forms. In this scenario, the unit, or “meta-organism” (Holmes and Moya 2002), can have a population-level structure that emerges from mutationselection dynamics, and the interacting variants can shape adaptive outcomes at the population level. Elevated mutation rates characteristic of viral replication machinery play a crucial role in the formation and maintenance of quasispecies, which allows for rapid adaptation to the host immune and antiviral responses (Domingo et al. 2012, Zhang et al. 2021, Chakraborty et al. 2022), enables escape from antiviral drugs and treatments (Svicher et al. 2011, Berkhout and Das 2012), transmission to new hosts (Wood et al. 2009, Xue and Bloom 2020), and colonization of new host tissues (Wood et al. 2009, Rife et al. 2016, Nijmeijer and Geijtenbeek 2019). Even within the same tissue, spatial organization can be important for virus cooperativity. For example, adeno-associated virus (AAV) has evolved to utilize a neighboring adenovirus helper during viral replication within a cell (Gonçalves 2005). Although AAV is able to infect cells on its own, the helper virus is required within the same cell for the production of AAV progeny (Meier et al. 2020). By sharing resources (e.g. replicase machinery), processes such as replication and encapsidation are not biased toward parental genomes, allowing even replication-incompetent viral genomes to be pack-aged and maintained in the population (Leeks et al. 2021). Though not capable of replication, these genomes can contribute WT and/or mutated genetic regions that can function in host adaptation and immune evasion (Vignuzzi and López 2019). Herpes virus, human papillomavirus, and vaccinia virus (Ronzitti et al. 2020) are additional viruses that exhibit co-dependence for enhanced replication and survival.

Given their rapid evolutionary rate and the predominantly deleterious nature of mutations, virus populations exhibit frequent compensatory genetic changes (Domingo et al. 2012). These co-evolutionary events can contribute significantly to disease development, immune evasion, and resistance to drugs (Berkhout and Das 2012, Chakraborty et al. 2022). As discussed in more depth in the next section, viral compensatory mutations are often studied in the context of individual fitness, but the role of mutational compensation at the population level in adaptation and survival is less investigated. In order to explore the evolutionary dynamics and persistence of pathogenic viral populations, it is essential to comprehend the potentially many types of linkage between genetic and phenotypic diversity and how these linkages affect viral-mediated disease (Domingo et al. 2012).

Understanding the distinctions between co-evolution, co-variation, and cooperation is essential for interpreting the next sections of this review. Co-evolution describes the synchronized evolutionary changes between two organisms or biomolecules, contributing to the preservation or enhancement of their functional interactions (Juan et al. 2013). Though used interchangeably in other settings, co-variation is not considered synonymous with co-evolution in this review. We instead refer to co-variation as the tendency of two or more traits or genes to vary together in a correlated manner across populations at frequencies exceeding random expectation (Armbruster and Schwaegerle 1996, Talavera et al. 2015). At a different level of biological organization, cooperation is again defined here as a behavior that enhances the fitness of another individual and may evolve because of the advantage it confers on the recipient(s) (West et al. 2007). Although these concepts are related, they are not interchangeable in this review. Co-variation and co-evolution can underlie both cooperative and competitive relationships, though only in cooperation do reciprocal changes increase collective fitness. This distinction is particularly important in a computational context, as some of these tools designed to detect co-evolutionary signals often identify correlated changes without being able to resolve whether such interactions are cooperative or competitive in nature. Consequently, clarifying these distinctions is key for interpreting how the tools and models reviewed here infer social mechanisms from molecular or evolutionary data.

## Co-variation and co-evolution as indicators of social behavior

The evolutionary biology community has recently shifted from studying independent variation of phenotypic traits to examining co-varying networks of traits across different biological levels (Armbruster et al. 2014), including micro-organisms. Since certain organisms need to maintain organizational unity for cooperation, modifications in one set of traits often give rise to corresponding adaptations in other traits that are interconnected through shared developmental pathways, functional activities, or genetic associations and pleiotropy (Armbruster et al. 2014). This inter-dependent relationship often produces measurable co-variation, ranging from readily observable traits, such as height, down to gene expression levels (Mackay 2009). Research on the variation of global transcription within and between groups via analysis of variance (Pletcher et al. 2002) has posited that if differences in gene expression contribute to variations in observable quantitative traits (Lafuente and Beldade 2019), correlated transcript levels can be used as proxies for inter-connected phenotypes (Pletcher et al. 2002). Yet, phenotypic variation and/or co-variation cannot always be explained by changes in gene expression.

An alternative approach to investigating phenotypic co-variation at the genetic level is through population genetics. Population genetic methods are used to estimate allele frequencies represented among and within populations (Falush et al. 2003) and to interpret how selection affects the configuration of genomic variation patterns (Hoffmann and Willi 2008, Joost et al. 2013). A population that exists with genotypic frequencies in equilibrium is defined by the Hardy–Weinberg Law (Hardy 1908, Weinberg 1908, Stern 1943) and sets a reference point to recognize and evaluate effects of selection, linkage, mutation, inbreeding, and chance (Mayo 2008). As described above, the emergence of mutations that lead to large phenotypic changes can disrupt this equilibrium (Stottmann et al. 2011, MacArthur et al. 2014, Renaut and Rieseberg 2015). Statistical and quantitative population genetic tools, such as those used in studying Hardy-Weinberg deviation (Gelman 2006, Kim et al. 2009, Friedman et al. 2010), are thus helpful for characterizing social dynamics within populations.

In the realm of viruses, a well-documented example of population-level compensatory mutation that can similarly act to rescue the community is that of the previously discussed H3N2 influenza virus. Two NA variants, D151 and G151, functionally complement each other. For populations containing strains for which HA exhibits reduced binding affinity (Lin et al. 2012, Gulati et al. 2013), a compensatory mutation in NA arises (D151G), restoring the lost receptor-binding function of HA (Gulati et al. 2005). As described above, this compensatory mutation does not become fixed (100%) within the population, but rather is kept at a steady state alongside the WT D151 variant. As a result, WT NA (D151) maintains its role in cell exit, while mutant NA G151 gains a new role in facilitating cell entry, enabling cooperative dynamics that increase population-level fitness (Xue et al. 2016).

As demonstrated above, co-variation can arise as a result of co-evolution (O and Whitlock 2023). However, the association of mutations with observed phenotypic changes is not always the result of compensatory change—it can be an intrinsic outcome of shared evolutionary history (phylogenetic connection) (Saputra et al. 2021). In other words, genes that are functionally related can undergo correlated selection pressures leading to genetic changes that are not necessarily independent (Saputra et al. 2021). Because of this, it is imperative to factor in analyses of the phylogenetic ties, or degree of evolutionary independence, among sampled individuals, particularly when seemingly codependent mutational events belong to the same species (Avila-Herrera and Pollard 2015). To illustrate, if species A and B appear to have acquired distinct mutations, but they are both descendants of the same ancestral lineage, both mutations could have actually arisen on the branch leading to the speciation event that resulted in the two individual species, rather than as a result of a needed compensation. This example demonstrates the interconnectedness of the mutations through the common ancestor, rather than co-evolution (Harrison et al. 1998).

Co-variation, or even co-evolution, does not always have to occur at the level of a single species. Evolutionary interaction between species was observed as early as 1862, when Charles Darwin deduced that to be able to feed from nectar, moths with a proboscis capable of extending inches in length must have co-adapted to a Madagascar orchid with a long, narrow nectar tube (Darwin 1892, Kritsky 1991). Over time, the concept of co-evolution expanded. It has grown to encompass a wide range of ecological interactions, including predator–prey relationships. Minute changes in the prey population can require that the predator population adjust accordingly, which can beget further prey adaptation, and so forth. This dynamic can lead to a range of observable phenotypes over the course of time (Mougi and Kishida 2009). Studies on the evolution of traits have since transitioned to focus, not just on interspecies interactions, but also on how the suitability of one or multiple species to its/their physical environment is influenced by genotypic changes (population genetic models) (Chacon et al. 2021). The merging of population genetics and predator-prey concepts has even been applied to explain viral behavior, as seen in the interaction between HIV and the immune system within the host (Rife Magalis et al. 2021). Massive organ sampling and viral data collection efforts within an animal (macaque) model of HIV infection revealed deterministic temporal fluctuations in the virus effective population size (*N_e_*), significantly correlating with immune cell populations at time lags indicative of a predator-prey system. In this system, the virus is hypothesized to continually adapt to evade the immune system, which in turn must alter the immune repertoire to target new variants (Rife Magalis et al. 2021). Hence, co-evolution is important in not only ecological maintenance, but also pathogen-host interactions (Gurney et al. 2017).

It is important to remember that, while co-evolution can be an indicator of phenotypic variation and cooperative interaction, the opposite is not true. Presence of allele frequency variation is not necessarily the result of a mutation, as there are other factors such as seasonal fluctuations in climate that can selectively favor particular alleles in different weather seasons, temperatures, or nutrient availability (Cvijovi et al. 2015). This occurrence highlights the intricate interaction between genetic and environmental factors in molding allele frequencies within populations, where mathematical modeling has gained increasing popularity in scientific research. These models present a framework for analyzing the processes through which cooperative behaviors may evolve and persist within populations, delving into the dynamics of altruistic and cheater actions within populations (Frey 2010).

## Theoretical models for evolution of cooperation

Understanding how cooperation arises through ecological, environmental, and/or evolutionary constraints has been approached through several complementary theoretical frameworks. In this section, we first describe classical mathematical and gametheoretic models that capture the strategic basis of cooperation and cheating. We then discuss ecological models, including the Black Queen hypothesis, that integrate community-level effects. Finally, we examine genome-scale and models that merge metabolic, ecological, and evolutionary perspectives. Together, these approaches illustrate how cooperation can be analyzed from abstract theory to mechanistic biological realism.

**Figure 2:**
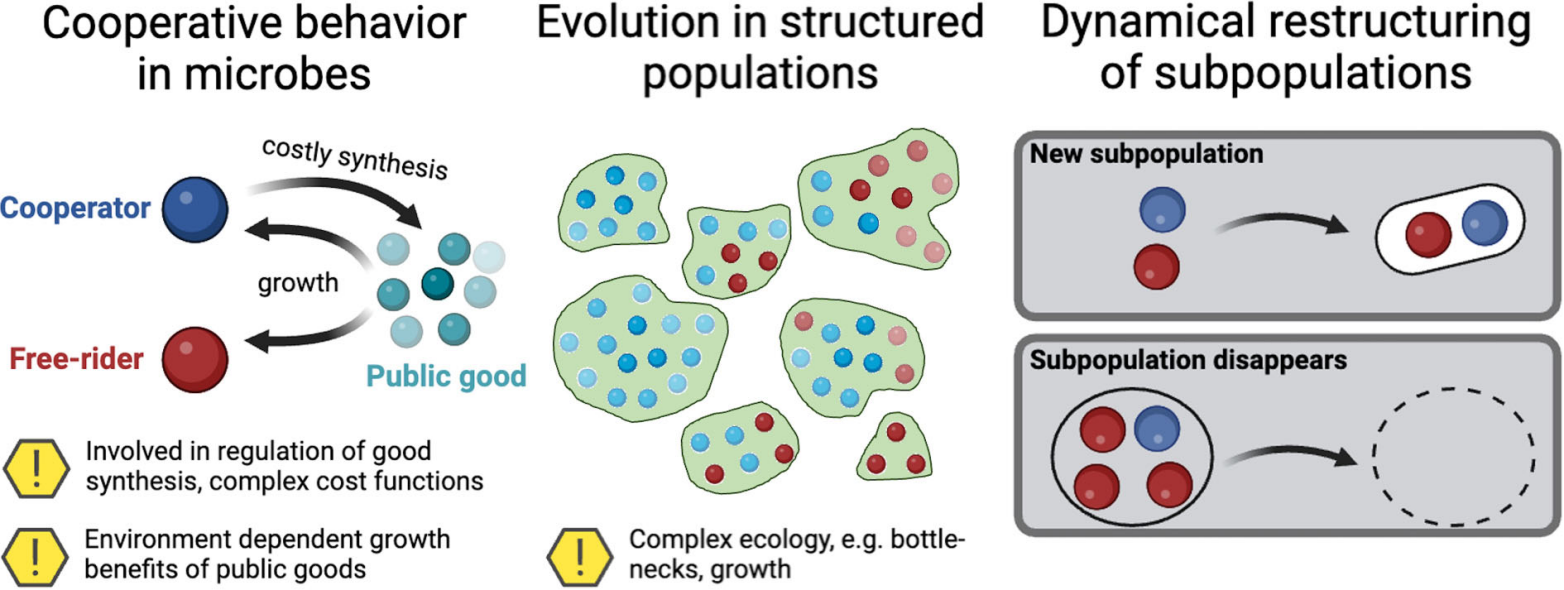
Microbial cooperation through production of public goods pits producers (cooperators) versus cheaters (free-riders) when adaptive gene loss of costly production induces obligate dependency on cooperators for growth [Black Queen (BQ) hypothesis]. Whether or not cheating overtakes all cooperative phenotypes in mathematical models depends on the formulation of frequency dependent selection. As observed in real microbial populations, the assortment of similar individuals (e.g. in biofilms) promotes cooperation via higher level selection that favors sub-populations of cooperators. Underlying metabolic processes, genotype-phenotype mapping and environmental factors can be added to models to further elucidate dynamic eco-evolutionary trajectories. Diagram adapted from (48).

Mathematical models have been used to understand the mechanisms by which cooperation can emerge, persist, evolve within a community, or even be overtaken by more selfish behavior. Evolutionary game theory is one method used to analyze the conundrum as to why individuals would produce costly public goods when cheaters can reap the benefits without loss. Within this context, certain conditions are needed for cooperation to be an evolutionarily stable strategy (a similar concept as a Nash equilibrium in game theory). For comprehensive reviews on the application of game theory to the evolution of cooperation, especially in microbes, see (Frey 2010, Hummert et al. 2014). Kin selection is often used to explain the emergence of cooperators, which can be described by Hamilton’s rule (Hamilton 1964)—a mathematical formula that supports the notion that natural selection favors genetic success, rather than reproductive success. Multi-level (or group) selection has also been considered in models of altruism (Nowak 2006). These two forms of selection can fit under a common mechanism for evolving cooperation, where there is some assortment of similar individuals (see Fig. 2).

The Black Queen (BQ) hypothesis is an ecological model used to describe cooperativity in the microbial world (Fig. 2), arguing that the exchange of essential metabolites between bacterial species co-evolves as adaptive gene loss of their costly production (Morris et al. 2012). This phenomenon could give rise to an obligate dependency of non-producers on producers, or, in the case of more than one BQ function, to obligate cross-feeding (bidirectional dependency) (Zomorrodi and Segrè 2017). Within kin, for example, cooperation goods tend to be passed to those individuals that carry the cooperative gene(s), however, when cheaters arise, cooperation benefits will be conveyed over time to the non-cooperative individuals, reducing the kin-selected advantages of cooperative interactions. This phenomenon in turn lowers the probability of favorable mutations to fix while increasing the likelihood of deleterious mutations (Oliveira et al. 2019, Belcher et al. 2022). Conceivably, these and similar dynamics can be studied in an evolutionary game theory model analytically when the set of strategies is small. Additionally, ecological models can allow for exploration of equilibria and stability in general networks of multi-species/resource phenomenological interactions, e.g. (Butler and ODwyer 2018, Marsland et al. 2019, Oña and Kost 2022). However, bacterial cooperation in the real world is often predicated on a complex network of metabolic interactions, along with the genetic architecture coding biochemical reactions which lead to phenotypic traits, which are not explicitly included in these models.

Genome-scale metabolic network models quantify a compilation of all intracellular biochemical reactions to offer a more fine-grained description of processes, not relying on implicit interaction terms of the models mentioned previously (Zomorrodi and Segrè 2016). Flux-based analysis (FBA), for example, is used to predict steady state fluxes (i.e. reaction rates) in the metabolic network by formulating the fluxes to be close to a predictable optimum (e.g. maximum biomass production), describing a state achieved by the cell through evolutionary adaptation (Perez-Garcia et al. 2016). This detailed approach has allowed for mechanistic modeling of microbial communities at the genome level, allowing model predictions to be directly compared with data to better understand microbiome assembly (Levy and Borenstein 2013) and diverse species interactions (Zelezniak et al. 2015). Related approaches have also been developed to infer cooperative interactions, such as *NetCooperate* (Levy et al. 2015), discussed in more depth in the next section. However, stoichiometric quantification of a large number of reactions complicates analysis of models; furthermore, genomic data often does not translate to functions needed in the metabolic formulation.

Merging the aforementioned modeling approaches can provide a unified framework for metabolic, ecological, and evolutionary dynamics of cooperation. Estimating game-theoretic payoffs with the FBA metabolic formulation in a genetic-structured ecological model has allowed for assessment of evolutionary stability of the BQ hypothesis when applied to epistatic and pleiotropic interaction in *E. coli* amino acids (Zomorrodi and Segrè 2017). The hybrid approach of genome-driven eco-evolutionary models informed by metabolic networks also can potentially be linked to phylodynamic simulation (Kühnert et al. 2016, Rasmussen and Stadler 2019, Lepers et al. 2021), which holds future promise for comparison with genomic sequence data. Similarly, network models of viral antigenic cooperation (Skums et al. 2015), wherein altruistic virus strains distract host immune response from persistent strains, may gain validity from phylogenetic tools to confront simulations with genomic data.

## Technological approaches to identify and measure cooperativity

Recognizing that bacteria and viruses are capable of social structures (Keller and Surette 2006) has driven the development of novel approaches to identify and measure cooperation (Buckling et al. 2007). Given that these micro-organisms have relatively short life spans, the study of evolution of cooperation in real time is more feasible than in, for example, vertebrate animals (Buckling and Brockhurst 2008). This research has been contributing significantly to several fields, including human (Rosenthal et al. 2011, Peters et al. 2012) and agricultural disease, biocontrol, crop growth and production (Baker et al. 1997, Berg et al. 2017, Carvalho 2018), and corrosion of oil and gas pipelines (Enning and Garrelfs 2014). As part of the investigation, it is necessary to gain insight into the mechanisms behind co-evolutionary dynamics and the resulting patterns of adaptation across populations (Ben-Jacob et al. 2000, Gurney et al. 2017). These interactions may be explained by sharing cooperative genes (Zaneveld et al. 2008) that display behaviors that can be mutualistic or altruistic among microbial communities that may aid in their reproductive success and fitness (West et al. 2006).

To illustrate how different computational frameworks capture mechanistic cooperative dynamics at multiple biological scales, we selected representative tools spanning metabolic, nucleic or amino acid sequence, and phylogenetic approaches (Table 1). Metabolic models such as *NetCooperate* and *RevEcoR* infer cross-feeding and metabolic dependencies, while co-evolutionary methods like *GREMLIN, MISTIC2*, and *AutoCoEv* model correlated evolutionary changes between residues or genes that may reflect functional interdependence. Other tools like *Mirror Tree* provide a phylogenetic framework for identifying correlated evolutionary histories between interacting proteins or species. Together, these tools exemplify how cooperation can be investigated through complementary computational and biological perspectives.

**Table 1:** Comparison of computational tools for identifying cooperation in biological systems.

Tool	Required data	Primary metric or output	Scope and applicability	Advantages and limitations					
				For large datasets?	Use in viruses?	Requires prior metabolic knowledge?	Requires advanced computing?	Analyzes pairwise interactions?	Analyzes network interactions?
NetCooperate	Metabolic pathways	Biosynthetic Support Score (BSS) & Metabolic Complementarity Index (MCI)	Cooperation and competition. Absence of complementarity may suggest competition	No	No	Yes	Yes	Yes	No
RevEcoR	Metabolic pathways	Competition index & Complementarity index	Cooperation (high complementarity index) and competition (high competition index)	No	No	Yes	No	Yes	No
GREMLIN	Multiple sequence alignment	Graph of conditional independencies among residues	Cooperation inferred from statistical dependencies among residues	No	Yes	No	Yes	Yes	Yes
MISTIC2*	Multiple sequence alignment	Mutual information (MI)	Cooperation inferred from correlated evolutionary signals	No	Yes	No	No	Yes	No
AutoCoEv	Phylogenies	Supported co-evolution events	Cooperation inferred from correlated evolutionary changes across phylogenies	Yes	Yes	No	Yes	Yes	No
Mirror Tree	Phylogenies	Supported co- evolution events	Cooperation inferred from correlated phylogenetic histories	No	Yes	No	No	Yes	No

The computational approaches reviewed below offer complementary and informative perspectives on biological interactions, each capturing distinct signatures of functional, metabolic, or evolutionary coupling. It is important to keep in mind that, while the individual methods alone cannot fully distinguish cooperative from competitive interactions, together they characterize a range of signals that can be used in integrative frameworks that combine mechanistic and ecological signals.

The principles underlying structured microbial communities have been the focus of ongoing discussion, as exploring the dynamics of microbial community formation and the interactions required is key to preventing pathogen cooperation and its harmful effects (Nemergut et al. 2013). Methodologies have been established to infer bacterial interaction networks by assuming that closely related species are more likely to coexist (Darcy et al. 2020), or, by analyzing the associations between small metabolites and the enzymes that facilitate various reactions within microbial systems (Bhattacharya et al. 2003). Some of these methodologies, described as early as 2010, continue to be used and remain relevant for characterizing cooperative mechanisms among well-known pathogens, and so below we describe the benefits of these methods. However, the focus of this review is the description of the limitations of these approaches for the expansion of the study of cooperative mechanisms to emerging pathogens, including viruses, as well as to microbes that may interact with an abundance of other species and for which the data are bountiful.

### Detecting cooperation using metabolic pathways

Originally, statistical and computational methodologies were created to postulate bacterial interaction networks using co-occurrence information (Friedman and Alm 2012, Mandakovic et al. 2018). These methods suffered from the frequent detection of indirect associations and use of parametric statistical models only (Hirano and Takemoto 2019). These strategies were slowly replaced by tools that focused primarily on metabolic pathways. These newer tools strive to deduce functional profiles or biological pathways from microbial co-evolving relationships; nevertheless, they present with a new set of challenges. Below, we describe the expressed function of these tools, their advantages in the realm of microbial cooperation detection, and their limitations as the research community attempts to use these tools to expand their field of view to incorporate emerging pathogens, including viruses.

#### NetCoopera*te*

NetCooperate is a web-based tool that builds on reverse-ecology tools (Janga and Babu 2008, Borenstein et al. 2008, Levy et al. 2015) to analyze metabolic networks and predict cross-species ecological interactions (Levy et al. 2015). It uses two-species metabolic networks as input, each represented as a directed graph with nodes denoting compounds and edges linking substrates to products. A seed detection algorithm identifies the “seed set”—a minimal group of compounds that must be taken from the environment and are required for biosynthesis—and evaluates the biosynthetic capacity of one species to fulfill the nutritional needs of another species. The resulting metrics include the Biosynthetic Support Score (BSS), which quantifies the ability of a host organism to fulfill the nutritional needs of a parasitic micro-organism, and the Metabolic Complementarity Index (MCI), which assesses biosynthetic complementarity between species. The associations identified by NetCooperate thus reflect functional coupling in a metabolic capacity indicating potential cross-feeding or mutual metabolic support, which may facilitate cooperative interactions.

To illustrate how NetCooperate can be used to investigate microbial interactions, one can adopt the framework used by Takemoto and Aie (2017) applied to two subpopulations of micro-organisms, A and B. Their approach used four metrics: biosynthetic support from A to B (*BSS_AB_*), biosynthetic support from B to A (*BSS_BA_*), the metabolic complementarity from A to B (*MCI_AB_*), and the metabolic complementarity of B to A (*MCI_BA_*) (Takemoto and Aie 2017). Cooperative interactions can be inferred when both *BSS_AB_* and *BSS_BA_* are positively associated, reflecting reciprocal exchange in which each subpopulation benefits from the other. Similarly, positive correlation for both *MCI_BA_* and (*MCI_AB_*) indicate that the two microbe subpopulations complement each other’s biosynthetic needs.

The advantages of NetCooperate are as follows: 1) BSS and MCI value implementation for both microbe-microbe and host-microbe interactions; 2) identification of nutrients needed to synthesize compounds in the network; and 3) the output log of the supported (or complemented) metabolites in each distinct species (Levy et al. 2015). Additionally, these types of approaches offer understanding of the natural biochemical habitat to explore the association and interaction of microbes with other organisms which may help identify symbiotic inter-species relationships, as well as give the possibility of predicting and designing drugs that affect an organism with no detrimental consequences for the host (Janga and Babu 2008).

On the other hand, some limitations of this tool include 1) advanced computational capabilities (Levy et al. 2015); 2) the need for prior knowledge of the metabolic pathways; 3) potential overestimation of false positives because of reversible reactions; 4) inconsideration of catabolic vs. anabolic pathways and potential alternative processes that regulate metabolic import (Janga and Babu 2008); not to mention 5) it can only analyze two organisms at a time. In the realm of mutualistic co-evolution, interest in research has recently expanded from the study of pairwise interactions to larger networks wherein species interact with several available partners (Bascompte and Jordano 2007, Guimarães et al. 2011, Guimarães et al. 2017), offering a more realistic perspective on pathogen survival *in vivo* that cannot be captured using dual-organism models. Considering an estimated 40%–60% of genes contribute an unknown function in microbial systems (Vanni et al. 2022, Rana et al. 2024), even a dual-organism model may be difficult for recently identified species with incomplete or unknown metabolic data representation (Levy and Borenstein 2013).

#### RevEcoR

**Figure 3:**
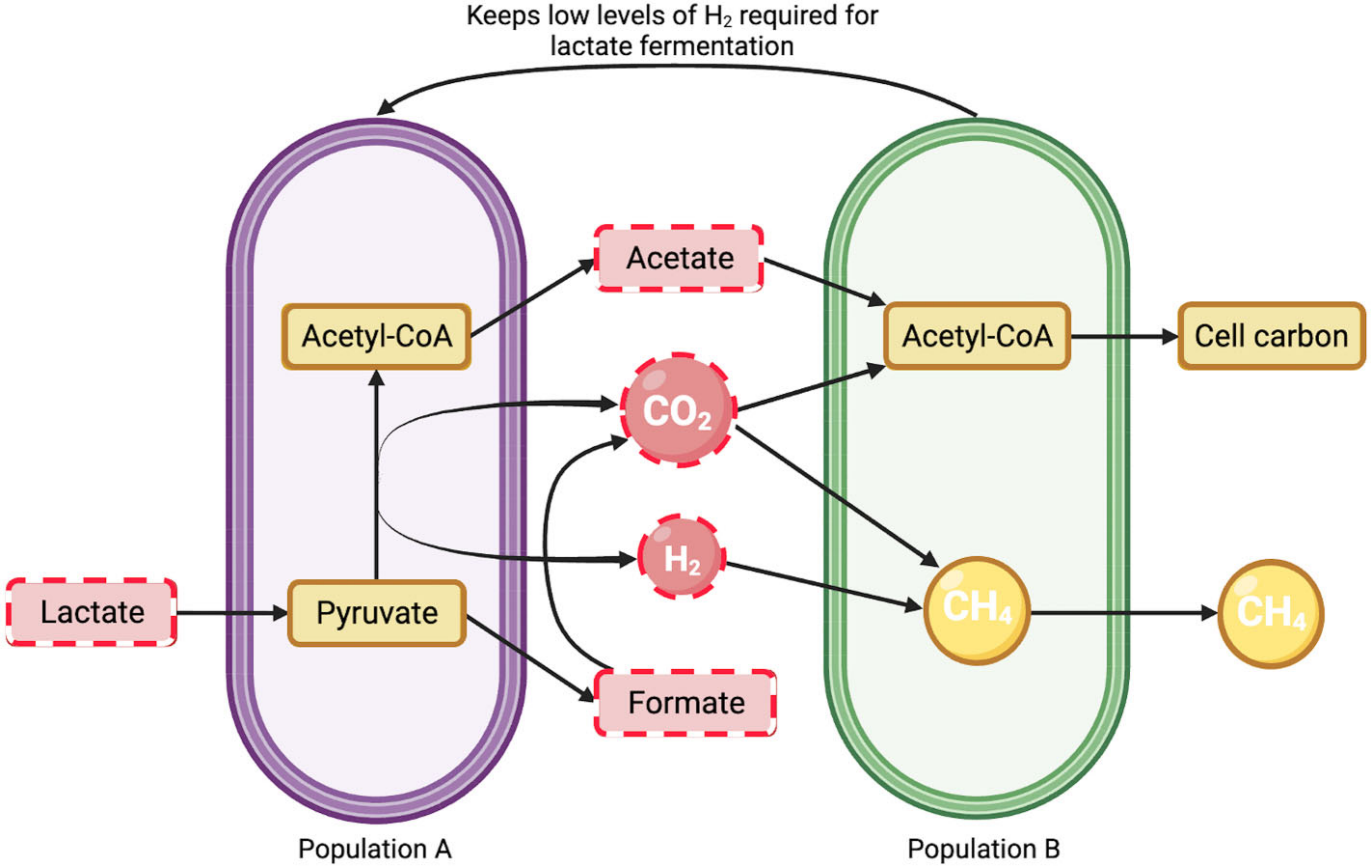
Schematic representation of metabolic networks that occur between two populations that allow the identification of cooperativity. Bacterial population A (left) ferments lactate into pyruvate and acetyl-CoA, producing acetate, CO, H, and formate (boxes/circles with dashed outlines, seed metabolites). Bacterial population B (right) consumes these intermediates to generate acetyl-CoA, cell carbon, and methane (boxes/circles with solid outlines). Inspired in the syntrophic relationship between *D. vulgaris* and *M. maripaludis* (Stolyar et al. 2007, Walker et al. 2012, Goyal et al. 2014).

RevEcor (Cao et al. 2016) is an R (R Core Team 2021) package that can be used to investigate multi-species interactions and predict their relationships in a particular environment. Like NetCooperate, RevEcor uses reverse ecology to reconstruct each species’ metabolic network. This tool uses functional annotated genomic data from Kyoto Encyclopedia of Genes and Genomes (KEGG) (Kanehisa et al. 2015) and the Integrated Microbial Genomes database (IMG) (Markowitz et al. 2013). A seed set algorithm (Borenstein et al. 2008) determines the set of compounds that are acquired from the environment by organisms (termed *seed set*) (Borenstein and Feldman 2009) and calculates two metrics—the competition index and complementarity index—for all pairs of microbes. The competition index represents the compound overlap in both species (metric of competition), whereas the complementarity index measures the compounds that are different between species (cooperation interaction). As with NetCooperate, these signals represent functional coupling derived from metabolic complementarity. A high complementarity index can suggest potential metabolic interdependence between species, which may be interpreted as ecological compatibility that could enable cooperative interactions. In the final metabolic network, directed graphs show nodes representing compounds and edges are metabolic reactions. A representative illustration can be observed in Fig. 3, where each species is depicted as a separate compartment. In this figure, the red nodes refer to the seeds and yellow nodes to the seed products of both micro-organisms, which are connected by directed arrows. In this example, the red nodes in the middle are the products from the first micro-organism that function as seed for the second micro-organism.

To evaluate potential cooperative interactions between microbial species, the authors of RevEcor, Cao et al. (2016) employed this tool on 116 prevalent gut species from a dataset with 124 samples. For each microbial pair present in the dataset, they obtained the complementarity index and the competition index from the tool, and also added a co-occurrence score. This co-ocurrence score was calculated using the Jaccard similarity index and reflected how frequently a pair of species were found together across this set of metagenomic samples. For example, the pair *E. coli* O157:H7 EC4115 and *Methanobrevibacter smithii* DSM 2375 exhibited a competition score of 0.313, suggesting a moderate overlap in required nutrients. However, their complementarity index was 0.563, pointing to a metabolic support between the species and potential co-operativity when they do occasionally co-exist (based on the low co-occurrence score of 0.135).

A shiny-based (Chang et al. 2024) interactive web application makes RevEcor accessible to users without advanced computational expertise. It is also reproducible, interactive, and can be launched in any R environment or remote server and is compatible with several platforms. It also supports high-throughput metagenomic data and uses the igraph R library (Csardi and Nepusz 2005) to store the metabolic networks, rendering it easy to use. Nevertheless, it comes with certain constraints: slow computational speeds, reliance on annotated metabolic data (Cao et al. 2016), potential inaccuracies and incompleteness due to noise in KEGG annotations in KEGG, all of which impact the reconstruction of metabolic networks.

It is important to note that for these metabolic-based tools, on top of requiring known metabolic networks, changes that occur in processes such as pathogenesis and other microbial interactions should be assessed in a dynamic setting as metabolism may change under different conditions and environments, particularly for bacteria. Additionally, the application of the above tools to viruses, which do not undergo metabolism, may not be feasible. Metabolic reactions of the infected cell may be considered proxies, though this has not been investigated. Given these disadvantages, genomic data may be considered more appropriate.

### Detecting cooperation using sequence data

State-of-the-art sequencing platforms are now readily available at affordable prices and consistently generate an immense volume of sequence data (Candela et al. 2015). Innovations in sequencing methodologies and instruments have also propelled rapid advancement in the genomic analysis of microbes (Rittmann et al. 2008); not to mention, it has provided the possibility of sequencing micro-organisms that have been unsuccessfully cultured and cannot be easily evaluated *in vitro* (Riesenfeld et al. 2004).

An advantage of using sequence data, especially for organisms with higher mutation rates, is that those genes involved in co-operativity show higher divergence and polymorphism rates. These polymorphisms can often be attributed to compensatory mutations associated with virulence factors or functions with large fitness consequences, such as social motility and nutrient uptake (Linksvayer and Wade 2009, Schuster et al. 2017, Belcher et al. 2022). As discussed above, co-evolution necessitates coordinated modifications of its constituents (Yeang and Haussler 2007). A most fundamental example occurs at the level of an individual macromolecule—nucleotide segments of an RNA molecule can interact to form secondary structures that function to regulate many different cellular processes (Chelkowska-Pauszek et al. 2021, Spitale and Incarnato 2022); mutations that significantly disrupt these structures often result in replication incompetence unless accompanied by compensatory mutations that act to rescue structure and function (Hancock et al. 1988, Pedersen et al. 2006, Knies et al. 2008, Wang et al. 2017, Zhang et al. 2019). The use of co-evolutionary information has similarly been applied to proteins in modeling their conformational dynamics and protein interactions, energetics, and folding rates (Morcos and Onuchic 2019). Ascending the molecular staircase, compensatory evolution can be essential to microbial survival, as exemplified above (see section on co-variation and co-evolution as a measure of co-operativity). Here, we introduce a non-exhaustive list of available tools that are often described as detecting co-evolution in nucleotide and protein sequences, although they primarily identify correlated sequence changes that may arise from shared selective pressures or functional constraints. We also outline how these tools have been, or can be, used to infer and/or characterize cooperation at the level of a microbial population.

#### Generative REgularized ModeLs of proteINs (GREMLIN)

GREMLIN uses a concatenated multiple sequence alignment as input for maximum likelihood estimation (MLE) of the parameters comprising a probabilistic graphical model with pair-wise log-linear potentials (Balakrishnan et al. 2011). The model output is a graph of protein residue interactions, which can range from disconnected to fully connected, depending on the chosen parameters for the regularization. The nodes in the graph represent the positions in the alignment, while the edges indicate which positions are conditionally dependent. Compared to other greedy algorithms, such as graphical models of residue coupling (GRMC) (Thomas et al. 2008), GREMLIN has a higher predictive accuracy.

In a study by Wellington Miranda et al. (2021), GREMLIN was used to identify co-evolving amino acid residues between acyl-homoserine lactone (AHL) synthases (LasI) and receptors (LasR) in quorum sensing (QS) systems. Although LasI and LasR do not interact physically, they are functionally related: LasI synthesizes AHL signals that can diffuse through cell membranes and are detected by LasR, which then regulates gene expression in a cell-density-dependent manner. These two proteins are thought to co-evolve to maintain recognition of a shared signal. To explore this, the authors analyzed synthase and receptor protein sequences from several bacterial genomes using GREM-LIN with average product correction (APC) to detect residues showing coordinated changes. The top-scoring co-evolving residues clustered around the ligand-binding pockets of both proteins. Since AHL signals can vary in their acyl chain length, saturation, and oxidation state, it was concluded that the observed coordinated changes likely result from evolutionary pressure to maintain communication specificity across chemically distinct signaling molecules. Experimental validation showed that single substitutions in these residues significantly altered signal production and detection. Interestingly, increasing LasR sensitivity to its cognate signal 3OC12-HSL often came at the cost of reduced selectivity, suggesting that natural QS systems may co-evolve to balance responsiveness and specificity. This balance appears to be critical to avoid premature activation of energetically costly social behaviors, such as toxin or protease production, which can ultimately lead to reduced biofilm fitness (Wellington Miranda et al. 2021). Overall, this study highlights GREMLINs ability to uncover co-evolution at the community level, even between non-interacting proteins to sustain cooperative signaling. However, it is important to note that co-evolutionary dependencies inferred by GREMLIN may reflect both physical and functional coupling. For the latter, it refers to residues or proteins whose activities that are interdependent. Interpreting these correlations as cooperation therefore may require additional biological context.

Graphical models of protein families have a central benefit of uncovering the direct and indirect interactions without generating robust inferences the inherent characteristics of the distribution across protein sequences. GREMLIN can analyze as few as 50 sequences to infer reasonable structures, and parallel processing across columns can optimize runtime. Its accuracy surpasses that demonstrated by DCA, and it can be further improved by incorporating a simple prior based on sequence separation and predicted secondary structure, particularly for alignments with only a few sequences (Kamisetty et al. 2013).

The drawbacks to GREMLIN concern 1) the lack of consideration of phylogenetic relationships (Balakrishnan et al. 2011) and 2) infeasibility for large datasets. When GREMLIN was initiated in an attempt to analyze the previously mentioned CadSpata5 protein pair, Petrov et al. revealed that a length of more than 1000 amino acids in an alignment are computationally demanding and may take prohibitively long to complete (Petrov et al. 2022).

#### Mutual information server to infer coevolution (MISTIC)

MISTIC is an interactive web server that estimates mutual information (MI) networks among proteins within a single, concatenated sequence alignment (Simonetti et al. 2013). The network generated is comprised of nodes (residues) connected by edges only when significant co-evolutionary signal is present. Higher MI values assigned to these edges indicate greater support for interacting residues or regions. Adjustments for entropy, phylogenetic bias, and data redundancy enhance the reliability of MI scores.

Overall, MISTIC has an interactive, user-friendly interface to evaluating protein family co-evolution. This tool has no limits for sequence length or number (Simonetti et al. 2013). However, when using hundreds of sequences, the application duration can be quite prolonged (two proteins can take 22 h of runtime), and interpreting large networks becomes challenging, making it unsuitable for resolving inter-molecular co-evolution (Petrov et al. 2022).

MISTIC2 (Colell et al. 2018), an update made to MISTIC, incorporates several co-evolution methods: Corrected Mutual Information (MIp), mean field direct coupling analysis (mfDCA), pseudo-likelihood maximization direct-coupling analysis (plmDCA), and multivariate Gaussian modeling DCA (gaussianDCA). Compared to MISTIC, MISTIC2 improves on compatibility and performance, offers extra information on residue annotations, and varied modalities for data filtering and figure exporting. However, during an attempted analysis of the Cad-Spata5 protein pair (Petrov et al. 2022), similar to DCA and GREMLIN, MISTIC2 crashed (Petrov et al. 2022). Furthermore, it requires the concatenation of the alignments when working with more than one protein, which can lead to confusion in interpreting co-evolution signals and differentiating between intra- and inter-protein relationships.

MISTIC2 can help to investigate cooperative interactions at the population scale by identifying statistically associated residues across a population. When protein sequences from a population of interest are concatenated, such as two interacting proteins, MISTIC2 computes mutual information (MI) for all residue pairs in the alignment. High MI scores indicate non-random coordinated changes, potentially arising from compensatory mutations, epistatic interactions, or shared selective pressures. Importantly, MISTIC2 extends beyond simple MI by incorporating more advanced inference methods such as MIp, mfDCA, and plmDCA. These allow for the distinction between direct and indirect interactions, improving the identification of functionally associated residue pairs. In the context of microbial populations, such co-evolving residues may reflect cooperation, wherein coordinated mutations maintain or enhance fitness under selective pressures, for example, host immunity or drug treatment. In this case, MISTIC2 infers statistical associations between residues—that is, correlated sequence modifications that are not necessarily coordinated—to identify potential cooperative interactions. However, while high MI values can reflect functional coupling or shared selective pressures, they can also arise from phylogenetic relatedness or epistatic constraints.

**Figure 4:**
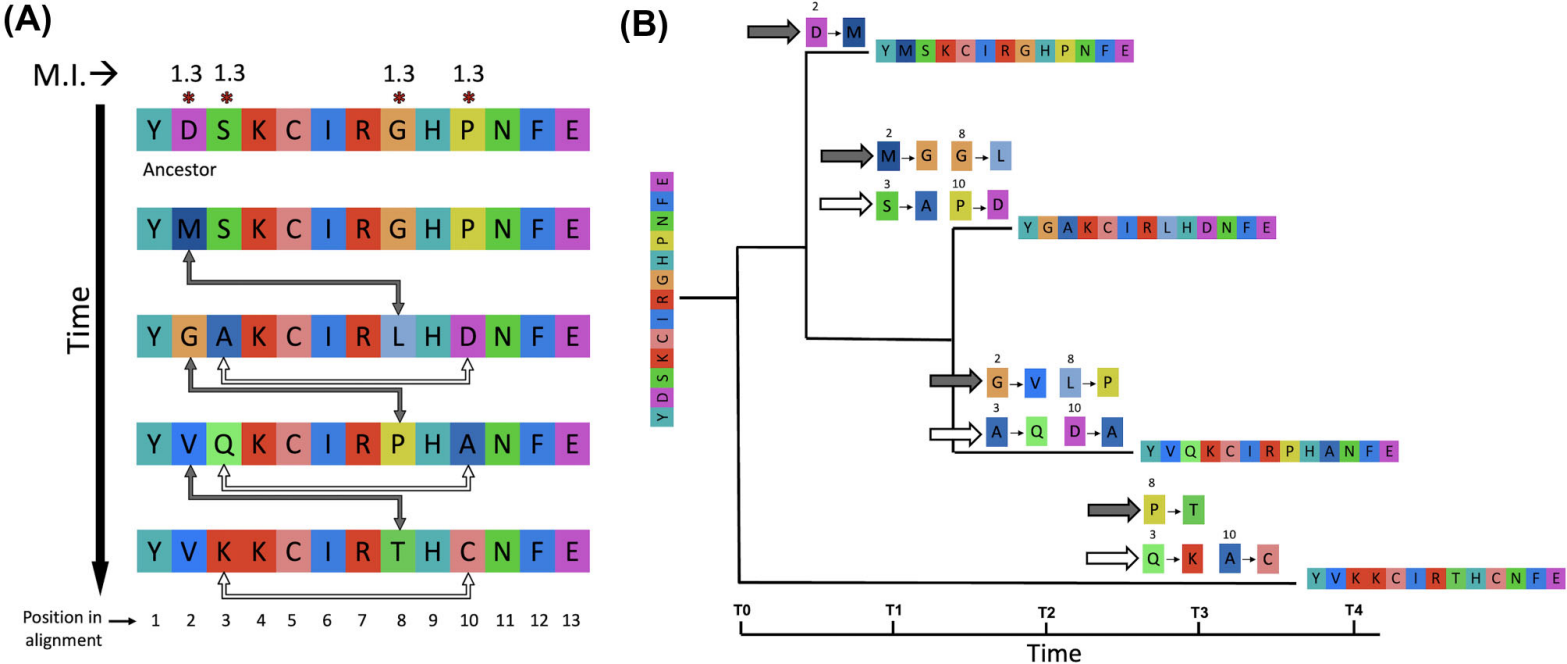
**(A)** Diagram of a multiple sequence alignment showcasing compensatory mutations over time at the population level (gray arrows) and individual molecular mutations (white arrows). Each row represents a distinct evolutionary stage, starting with the ancestral sequence displayed at the top. Distinctly colored letters denote different amino acids. Asterisks located at the top of the figure highlight the sites that exhibit changes, with mutual information values calculated in the Entropy-One web tool from the Los Alamos National Laboratory (Los Alamos National Laboratory, 2021). **(B)** Phylogenetic tree corresponding tosequences in **(A)**, illustrating the compensatory mutations observed. Amino acid changes over time are mapped onto their respecting branch with arrows indicating mutations at either the population (gray arrows) or individual (white arrows) level, as shown in **(A)**.

All of these tools that rely primarily on alignments for co-evolution analysis may detect correlated changes; however, sequence evolution is governed by fundamental molecular interactions encompassing both intra-molecular connections within protein or RNA structures and inter-molecular interactions in protein complexes and signaling pathways (Avila-Herrera and Pollard 2015). These associations between the patterns of substitutions in the columns of multiple sequence alignments may also be detected as such as a consequence of epistasis (Avila-Herrera and Pollard 2015) (Fig. 4, white arrows). Specifically, patterns of substitutions can be found using mutual information, which quantifies the relationship between two random variables (Tsalatsanis et al. 2020). An example is depicted in Fig. 4, where mutual information is used to delineate the degree to which information observed at one amino acid position (or column) aids in forecasting another position (Martin et al. 2005). As can be seen in this figure, MI values are the same for intra-protein and inter-protein residue interactions (Fig. 4A), which are more easily discerned in the context of phylogenetic relationships among the sequences (Fig. 4B). Likewise, co-evolution or independent evolution can both result in similar patters of coordinated changes between traits. This similarity adds uncertainty in distinguishing whether traits evolved together due to co-evolution or independently due to other factors. (Talavera et al. 2015, Avila-Herrera and Pollard 2015). Detecting physically interacting sites often requires broad and deep alignments, often ranging from hundreds to thousands of sequences (Avila-Herrera and Pollard 2015), power that most of these tools are lacking.

### Detecting cooperation using phylogenies

Current phylogenetic methodologies allow for the reconstruction of ancestral molecular sequences for related individuals (Jowkar et al. 2022). Through the examination of both ancestral and descendant sequences, it is possible to trace compensatory mutations over time (Szamecz et al. 2014) and to distinguish mutations that are correlated through true co-evolution from those that appear correlated through shared ancestry. Below we describe tools that consider phylogenetic relationships, as well as purely phylogeny-based methods.

#### AutoCoEv

AutoCoev comprises an automated command-line workflow that uses as many as 15 programs for the screening of correlated evolutionary changes within a multi-protein sequence alignment and/or phylogeny, with CAPS2 [coevolution analysis using protein sequences (Fares and McNally 2006)] as the center of the pipeline (Petrov et al. 2022). Input includes a list of proteins with their UniProt (Consortium et al. 2022) identifiers, a list of species for ortholog search, or a phylogenetic tree. Using OrthoDB (Kuznetsov et al. 2022), AutoCoev looks for homologous proteins for the species/proteins of interest, and each UniProt ID is matched to its corresponding OrthoDB ID. Using a program called Guidance (Sela et al. 2015), orthologs that may be too divergent are excluded, which can affect the robustness of the alignment. In the last step of the pipeline, CAPS2 detects co-evolution among individual protein pairs. The final output consists of a list of all co-evolving amino acid pairs which can be visualized in Cytoscape (Petrov et al. 2022).

AutoCoev can be adapted to investigate cooperation at the population level by detecting co-evolutionary relationships between proteins that interact or functionally complement each other across individuals. The pipeline, available on GitHub (https://github.com/mattilalab/autocoev), allows users to specify the species of interest and adjust alignment quality thresholds using the Guidance program. For example, setting the Guidance cutoff to 0 disables the exclusion of divergent sequences, which may be desirable when capturing variation across diverse populations. AutoCoev then screens for statistically significant correlated mutations between protein pairs using CAPS2, potentially revealing coordinated residue changes indicative of functional co-adaptation. In addition, the user can define protein pairs of interest in an optional list, facilitating the detection of co-evolutionary signals in cross-species interactions, such as metabolic cooperation or molecular symbiosis. It should be noted that the co-evolutionary relationships identified by AutoCoev mainly capture functional coupling among residues or proteins, reflecting coordinated substitutions that preserve structural or biochemical integrity and can sometimes be interpreted as cooperative interactions.

Among its advantages, AutoCoev allows users to specify a list of species and a list of protein pairs, can achieve batch processing of hundreds of proteins while also preventing biased results from file upload order by running CAPS twice (i.e. detected co-evolving protein pairs are included in reverse order in the second run). However, with a heavy focus on mutations involved in co-evolution, AutoCoev is prone to overlooking interactions within deeply conserved protein domains (Petrov et al. 2022).

#### Mirror tree

Mirror Tree is an automated for co-evolutionary analysis between two protein families and examines the likelihood of interactions and functional correlations within a taxonomic context (Ochoa and Pazos 2010). Input options range from two sequences to two alignments or phylogenetic trees, that will be locally aligned [BLASTed (Altschul et al. 1997)]. For single sequences, orthologs are searched against the Integr8 database (Kersey et al. 2004), filtered to exclude any fragmentary and divergent sequences, and alignment of the remaining sequences with MUSCLE (Edgar 2004). A phylogenetic tree is generated from each of the final alignments using the neighbor-joining (NJ) (Saitou and Nei 1987) algorithm implemented in CLUSTALW (Chenna et al. 2003). Mirrored branching patterns within the two trees are considered evidence of similar or linked evolutionary pathways, which may be indicative of cooperative interactions.

This approach was used by Yin and Yau (2017) to investigate co-evolution among all seven proteins encoded by the Ebola virus genome (GP, NP, VP24, VP30, VP35, VP40, and L) using 75 complete Ebola virus genomes. Each protein sequence was aligned using Clustal Omega, and phylogenetic trees were constructed for each set using the Mirror Tree server. This study revealed consistently high Pearson correlation coefficients, particularly between NPVP30 (0.987), VP24VP40 (0.979), and VP30VP40 (0.968), suggesting strong co-evolutionary relationships across the population, where these proteins may originate from different viral particles. (Yin and Yau 2017). Functional roles of these proteins support these findings: NP and VP30 may cooperate in transcription, while VP24 and VP40 are involved in virion assembly and potentially transcriptional regulation. These patterns point to molecular cooperation, where compensatory evolution preserves essential physical and regulatory interactions critical to the viral life cycle.

Among the major advantages of Mirror Tree is it is user-friendly, there is no requirement for providing a previously generated alignment and/or phylogeny. On the other hand, it does allow for proficient users to upload trees reconstructed from alternative methods (e.g. maximum likelihood). Some of Mirror Tree’s disadvantages include 1) generalizing co-evolution to entire proteins rather than individual domains and 2) full-length sequences are needed as input when starting with an alignment (i.e. missing genetic information is not permitted). Additionally, one user is only permitted to submit one job every 10 minutes to avoid server overload (Ochoa and Pazos 2010).

#### Bayesian graphical modeling

Bayesian graphical modeling (BGM) is a probabilistic graphical model originally used to model intricate data structures (Ni et al. 2021) that have become attractive for biomedical analysis because they consider conditional dependence and the accompanying uncertainties (Ni et al. 2018). Unlike many co-evolution metrics, the lack of phylogenetic information (Yeang and Haussler 2007), BGM implementations compute the joint posterior probability distribution of parameters within phylogenetic models (Höhna et al. 2016). The sequences manifesting the same level of coordinated changes may arise from either a few independent substitutions in early ancestors or correlated changes along the same lineage multiple times over the course of evolutionary history (Yeang and Haussler 2007).

BGM was applied to HIV-1 subtype B protease to identify statistically robust patterns of coordinated amino acid substitutions, a protein with well-documented structural and functional roles in compensatory mutation and drug resistance (Poon et al. 2007). Over two thousand HIV-1 protease sequences were analyzed from patients treated with antiretroviral therapy (ART) that included at least one protease inhibitor. A maximum-likelihood phylogeny was inferred and ancestral state reconstruction was performed. By comparing ancestral codons reconstructed along individual tree branches, non-synonymous substitutions were encoded in a binary matrix, with rows representing branches and columns representing codon sites. The resulting matrix was used to infer a Bayesian network in which nodes represented codon sites and directed edges captured conditional dependencies indicative of co-evolution. A threshold of 0.95 on marginal posterior probabilities was applied, producing a consensus network of 16 co-evolving site pairs (Poon et al. 2007). However, in this specific case, the resulting network of co-evolutionary relationships were limited to substitutions occurring along individual branches, reflecting individual-level co-evolution. Future analyses could extend this framework to detect co-evolving sites across multiple branches, revealing population-level functional dependencies.

An example of BGM is SPIDERMONKEY, (Poon et al. 2008), a free web-based phylogenetic tool that detects co-evolving sites from a nucleotide or protein multiple alignment. It uses maximum likelihood techniques to reconstruct ancestral sequences and applies BGM to determine significant relationships between sites in the sequences (Pond et al. 2004). The tool outputs a graph of nodes connected by directed edges representing conditional dependence between sites in the sequences. It is available in the HyPhy (Kosakovsky Pond et al. 2019) software package and Datamonkey webserver (Weaver et al. 2018). The web version restricts alignments to 500 sequences and 1000 codons at most (Avino and Poon 2018). This poses a major limitation as large genes over 1000 codons (Karlin et al. 1998) could be implicated and interactions like cooperation usually involve multiple genes (Wilson and Filipp 2018). Due to constrained computing resources, it also limits the amount of conditional dependencies to one or two per node and restricts the number of steps that the Markov chain Monte Carlo sampler can run. Nevertheless, the authors mention that these steps are sufficient for convergence, with customization options via additional resources and scripts (Avino and Poon 2018). Importantly, however, the search for co-evolution is limited to parent and child nodes (i.e. a single branch) within the phylogeny in SPIDERMONKEY, restricting its scope to epistatic interactions and/or individual-level fitness inferences (Kryazhimskiy et al. 2011), rather than population-level (see Fig. 4B). The co-evolutionary relationships identified by BGM primarily represent functional coupling between sites, accounting for shared evolutionary history through its phylogenetic framework. Such networks can reveal molecular interdependencies that support cooperative traits through compensatory mutations.

Advantages of using BGM include managing incomplete data, which prevents the over-fitting of data and obtain the causal relationships among sites (Poon et al. 2008). BGM reduces false positives by accounting for background rate variation among branches and integrates machine learning and phylogenetic methods to address the identity by descent of shared genotypes (Poon et al. 2008). This makes BGM more robust compared to other approaches such as MI statistics which lack parametric properties (Dimmic et al. 2005). BGM networks are, therefore, considerably more advantageous when compared to pairwise association analysis (see Detecting cooperation using metabolic pathways and Detecting cooperation using sequence data sub sections).

## Concluding remarks

Similar to multi-cellular organisms, micro-organisms such as bacteria and viruses can participate in intricate cooperative inter-actions that contribute to their social nature. While the processes behind microbial cooperation are not entirely clear, they are likely driven by the benefits of living in groups, including greater resource availability and increased protection from environmental challenges, not so different from the animal kingdom. These cooperative relationships can facilitate the evolution of pathogenic characteristics (e.g. immune evasion) and the ability to compensate for mutation-driven deficits that would otherwise compromise the population (e.g. cheaters). There has been a disparity in the computational models employed so far in terms of the ability to identify cooperativity and the pathways involved, the cause of which is multi-faceted. First, many of these existing tools rely on additional *a priori* data (e.g. already known and characterized metabolic pathways), which restricts the ability to identify novel interactions in even commonly studied micro-organisms, but also for poorly characterized micro-organismal populations such as recently emerging pathogens. Importantly, increasing evidence points to an important role for cooperation in critical virological functions (e.g. immune evasion), but viruses do not actively metabolize substances, as they rely on host cells to carry out replicative functions. Hence, metabolic pathways as they are defined for bacteria cannot be used in this way. One alternative here is to provide data for the cell infected by the virus, which is known to be influenced by the presence of the virus (Li et al. 2023).

Given the availability of sequencing technology and data, many developers and researchers have turned to genomics as a source of information on co-variation, which has the benefit of being able to be collected and monitored in realistic environmental settings (e.g. *in vivo* samples). Co-evolution within genomic data can be used to identify co-variation among different genes or proteins and has proven indispensable for studying the maintenance of stable micro-organismal communities. Different genomic features and evolutionary patterns serve as indicators of co-evolution, resulting in numerous available computational tools discussed in this review. However, the rise of high-throughput sequencing and emerging evidence of multi-species interactions has not yet witnessed the necessary increase in capability of genomic tools to handle large datasets. Moreover, only a few of these methods consider phylogenetic relationships, either as a contributing factor to false positive interactions or as a way to distinguish individual-level interactions from population-level interactions. Co-evolutionary events detected across branches of a phylogeny during a reasonable window of time would be advantageous for identifying novel interactive gene pathways within microbial populations for which metabolic networks are unknown or incomplete or even populations for which social behavior has not yet been described.

As with many of the co-evolution methods discussed in this review, one limitation of a phylogeny-based approach would remain—co-evolution does not necessarily indicate cooperation, as antagonistic interactions, or competition, are also rep resented by compensatory mutations. This decoupling challenge is evident across many of the computational approaches discussed herein. For example, RevEcor quantifies metabolic complementarity and competition indices to infer potential cooperative or competitive interactions at the level of inferred metabolic potential. However, because it relies on static network reconstructions and does not truly capture the dynamics of resource exchange, it cannot definitively determine whether inter-actions between micro-organisms are realized as cooperative under some conditions and competitive under others. Relatedly, NetCooperate infers the potential for cooperation through measures of biosynthetic support and complementarity, but low complementarity alone does not necessarily imply competition and may instead reflect metabolic independence or potential incomplete pathway annotation. A suggested step forward in the support of genomic studies of cooperation is, therefore, the scaling and integration of complementary approaches. Mathematical ecological models, as described in this review, may provide the missing link by translating these static or correlative signals into more context-dependent fitness outcomes, thereby enabling the decoupling of cooperative and competitive interactions that arise from the same underlying dependencies. In this way, phylogenetic models capture long-term shared evolutionary histories with ecological or dynamical models determining whether these interactions increase or decrease fitness under the environmental conditions of interest. With the incorporation of phylogenetic and ecological models with cellular and metabolic data across bacterial and viral systems, it becomes possible to develop a unified framework for mechanistic and causal inference into the dynamics of pathogen social interactions *in vivo*. By drawing together insights from these approaches, this review illustrates how current tools can be interpreted through the lens of microbial cooperation, while outlining areas where further development is needed.
